# Fine Mapping of Lycopene Content and Flesh Color Related Gene and Development of Molecular Marker–Assisted Selection for Flesh Color in Watermelon (*Citrullus lanatus*)

**DOI:** 10.3389/fpls.2019.01240

**Published:** 2019-10-08

**Authors:** Chaonan Wang, Aohan Qiao, Xufeng Fang, Lei Sun, Peng Gao, Angela R. Davis, Shi Liu, Feishi Luan

**Affiliations:** ^1^Key Laboratory of Biology and Genetic Improvement of Horticulture Crops (Northeast Region), Ministry of Agriculture, Northeast Agricultural University, Harbin, China; ^2^College of Horticulture and Landscape Architecture, Northeast Agricultural University, Harbin, China; ^3^Woodland Research Station, Sakata Seed America, Inc. Woodland, CA, United States

**Keywords:** lycopene, gene mapping, flesh color, CAPS markers, marker-assisted selection (MAS)

## Abstract

Lycopene content and flesh color are important traits determined by a network of carotenoid metabolic pathways in watermelon. Based on our previous study of genetic inheritance and initial mapping using F_2_ populations of LSW-177 (red flesh) × cream of Saskatchewan (pale yellow flesh), red flesh color was controlled by one recessive gene regulating red and pale yellow pigmentation, and a candidate region related to lycopene content was detected spanning a 392,077-bp region on chromosome 4. To obtain a more precise result for further study, three genetic populations and a natural panel of 81 watermelon accessions with different flesh colors were used in this research. Herein, we narrowed the preliminary mapping region to 41,233 bp with the linkage map generated from F_2_ populations of LSW-177 (red flesh) × cream of Saskatchewan (pale yellow flesh) with 1,202 individuals. Two candidate genes, *Cla005011* and *Cla005012*, were found in the fine mapping region; therein *Cla005011* was a key locus annotated as a lycopene β-cyclase gene. Phylogenetic tree analysis showed that *Cla005011* was the closest relative gene in gourd. LSW-177 × PI 186490 (white flesh) and another BC_1_ population derived from garden female (red flesh) × PI 186490 were generated to verify the accuracy of the red flesh candidate gene region. By analyzing the expression levels of candidate genes in different developmental stages of different color watermelon varieties, *Cla005011* for the expression differences was not the main reason for the flesh color variation between COS and LSW-177. This indicated that the *LCYB* gene might regulate fruit color changes at the protein level. A new marker-assisted selection system to identify red and yellow flesh colors in watermelon was developed with flesh color–specific CAPS markers and tested in 81 watermelon accessions.

## Introduction

The watermelon (*Citrullus lanatus*) is one of the most important cucurbitaceous crops in the world and occupies approximately 6% of the cultivated area used for all types of vegetables. The main pigment that causes red flesh color in watermelon is lycopene, which is considered one of the most important natural carotenoids in fruits. Lycopene has been a research focus in many areas, including health care products, cosmetics, and nutrition, and has been shown to serve physiological functions in the human body. Lycopene is also the precursor in some physiological, and biochemical processes in plants. Free radical scavenging can damage DNA and proteins, and one effective treatment is high lycopene intake (Mortensen et al., 1997). Cancer prevention, immunity enhancement, and cardiovascular protection can also be improved by lycopene (Feng et al., 2010). Notably, the lycopene in watermelon can be absorbed by the human body directly, but for tomato, another fruit rich in lycopene, dietary lycopene is better absorbed from cooked foods. Lycopene is also the precursor of β-carotene, violaxanthin, and neoxanthin, which participate in different physiological events in plants, including photosynthesis, antenna assembly, and photoprotection (Young, 1991; Latowski et al., 2011).

The visual quality of flesh color in watermelon is an important commodity characteristic for consumers, and different compositions of carotenoids create a wide range of watermelon flesh colors. The main flesh colors for watermelon are white, pale yellow, canary yellow, orange, pink, red, and scarlet. Furthermore, some accessions belonging to the subspecies *Citrullus amarus* are light green. Only trace amounts of phytofluene could be detected in white-fleshed watermelon (Zhao et al., 2008). In watermelons with yellowish flesh, the pigments were found to be a mixture of β-xanthophyll derivatives generated from zeaxanthin, but the composition was different in different accessions. Neoxanthin, violaxanthin, and neochrome were the three main pigments (1.66 and 0.29 µg/g for canary yellow and pale yellow, respectively) (Bang et al., 2010), while all-*trans*-violaxanthin, 9-*cis*-violaxanthin, and luteoxanthin were detected in yellow-fleshed watermelon by Liu (2.01–2.82 µg/g) (Liu et al., 2012). Watermelons with orange flesh had much higher contents of ζ-carotene, prolycopene, or β-carotene than of other pigments (Tadmor et al., 2005). The main pigments found in pink, red, and scarlet watermelon germplasm resources were lycopene, trace amounts of phytoene, prolycopene, and xanthophyll (Perkins-Veazie et al., 2006), while ζ-carotene, zeaxanthin, violaxanthin, and other carotenoids were barely detectable in mature red fruit (Grassi et al., 2013).

The inheritance of red, yellow, and white flesh color in watermelon has been previously reported. Canary yellow (*C*) is dominant to most other flesh colors (*c*), such as red, pink, orange, and pale yellow except white, which was designated *Wf*. The *Wf* gene is epistatic to the yellow flesh trait. Henderson et al. (1998) reported the allele *i-C* (inhibition of *C* and *c*), which was epistatic to *C* and generated red flesh even in the presence of *C*, but these results were not validated by Bang et al. (2010), who studied the genetic basis of red and canary yellow flesh colors in watermelon. In addition, the *py* gene resulting in pale yellow flesh was reported by Bang et al. (2010).

Some major genes or QTLs related to flesh color in cucurbitaceous crops (such as watermelon, melon, and cucumber) have been reported by scientists. In watermelon, the first QTL mapping of flesh color was performed by Hashizume et al. (2003), and two QTLs related to red flesh color were mapped on LG II and VIII with an integrated genetic linkage map. In our previous study, a major QTL (*LCYB 4.1*) conferring lycopene content and red flesh color was located in a 392,077-bp region on chromosome 4 based on a genetic background including red and pale yellow flesh colors (Liu et al., 2015). *ClPHT4;2* were identified to regulate the development of the flesh color through the process of the carotenoid accumulation. In cultivated watermelon varieties, the transcription level of *ClPHT4;2* was controlled by transcription factors *ClbZIP1* and *ClbZIP2*. And the *ClPHT4;2* not only regulate flesh color, but also could control the level of sweetness (Zhang et al., 2016). Subsequently, Branham et al. (2017) had mapped one major gene associated with β-carotene accumulation as regulating the color change of the fruit in watermelon by one yellow fruited line from a cross of NY0016. In melon, a locus associated with β-carotene was detected on chromosome 9 with a high-density linkage map by Harel-Beja et al. (2010). Based on this finding, Tzuri et al. (2015) found that the sequence of the *CmOr* gene in this effective region was homologous to *BoOr* in cauliflower, which controls the accumulation of β-carotene (Lu et al., 2006). Six single-nucleotide polymorphisms (SNPs) differed between the homologous sequences of *CmOr* in orange- and green-fleshed accessions, with only one functional variation (G base to A base) that changed arginine to histidine at the 323rd amino acid position in melon. The expression of *CmOr* was not significantly affected by growth stage in green- and orange-fleshed melons according to reverse transcription–polymerase chain reaction (PCR), implying that the allelic variation in *CmOr* did not affect β-carotene accumulation by altering the transcript level. The same results were also observed in an RNA-seq bulk analysis: most of the genes participating in the carotenoid metabolic pathways exhibited no significant expression changes between the green- and orange-fleshed melon bulks, even though the β-carotene content was significantly different in these two bulks (Chayut et al., 2015). β-Carotene could be detected in callus when the allele of the homologous *CmOr* gene associated with green flesh (arginine into histidine) was transformed into *Arabidopsis* by Tzuri et al. (2015). The F_3_ population was obtained by hybridization of white and orange flesh melons, 131 plants were genotyped by RAD-seq, and a major white flesh QTL locus (*CmPPR1*) was found on chromosome 8 (Galpaz et al., 2018). In cucumber, the *ore* gene, which was located on cucumber chromosome 3DS using a RIL (recombinant inbred lines) population, was the key gene for β-carotene accumulation (Bo et al., 2011). Pale yellow flesh color in cucumber was affected by a single recessive gene, *yf*, and fine mapped to a 149.0-kb region on cucumber chromosome 7 containing 22 candidate genes (Lu et al., 2015).

Compared with conventional breeding, rapid breeding through the marker-assisted selection (MAS) method is considered to be an effective strategy for the development of various cultivars. Thus, the investigation of genetic patterns, major gene locations, and specific markers associated with desirable traits should be top priorities for breeding programs. As relatively few studies of lycopene content and flesh color beyond those cited above have been performed in watermelon, this topic deserves further study. In the present study, we narrowed the lycopene content and flesh color–related QTL region with two genetic populations and investigated the candidate genes. The results provide a research foundation for candidate gene functional validation and support the development of breeding strategies that incorporate traditional and molecular approaches for developing accessions with different flesh colors and MAS in watermelon flesh color breeding.

## Materials and Methods

### Plant Materials

Two red-fleshed lines, “LSW-177” and “garden female” (*C. lanatus* subsp. *vulgaris*); one pale-yellow–fleshed line, “cream of Saskatchewan,” abbreviated “COS” (*C. lanatus* subsp. *vulgaris*); and one white-fleshed line, “PI 186490” (*C. lanatus* subsp. *mucosospermus*), were used in our research for genetic population construction. The seeds of LSW-177, COS, and PI 186490 were kindly provided by ARD from the US Department of Agriculture, Agricultural Research Service, South Central Agricultural Research Laboratory. The seeds of garden female were provided by the Laboratory of Molecular Genetic Breeding in Melon and Watermelon, Horticulture College of Northeast Agricultural University, China. Three hybrid combinations segregating for fruit flesh color (LSW-177 × COS, LSW-177 × PI 186490, and garden female × PI 186490) were performed to produce the genetic populations. For the LSW-177 × COS and LSW-177 × PI 186490 crosses, the F_1_ plants were self-pollinated to produce two F_2_ generations (Pop. 1 and Pop. 2) including 352 and 359 individuals, respectively. Meanwhile, we increased population size to 1,202 plants of the same source as Pop. 1 for fine mapping. For the garden female × PI 186490 cross, the F_1_ plants were backcrossed to garden female to obtain a BC_1_ population (Pop. 3, 222 plants). These three genetic groups were used to describe and validate the inheritance patterns of the flesh color and lycopene content traits in mature fruits. Pop. 1 and Pop. 2 were used in genetic map construction and linkage analysis for gene mining and candidate region reduction; Pop. 2 and Pop.3 were also used to verify the mapping results.

**Figure 1:**
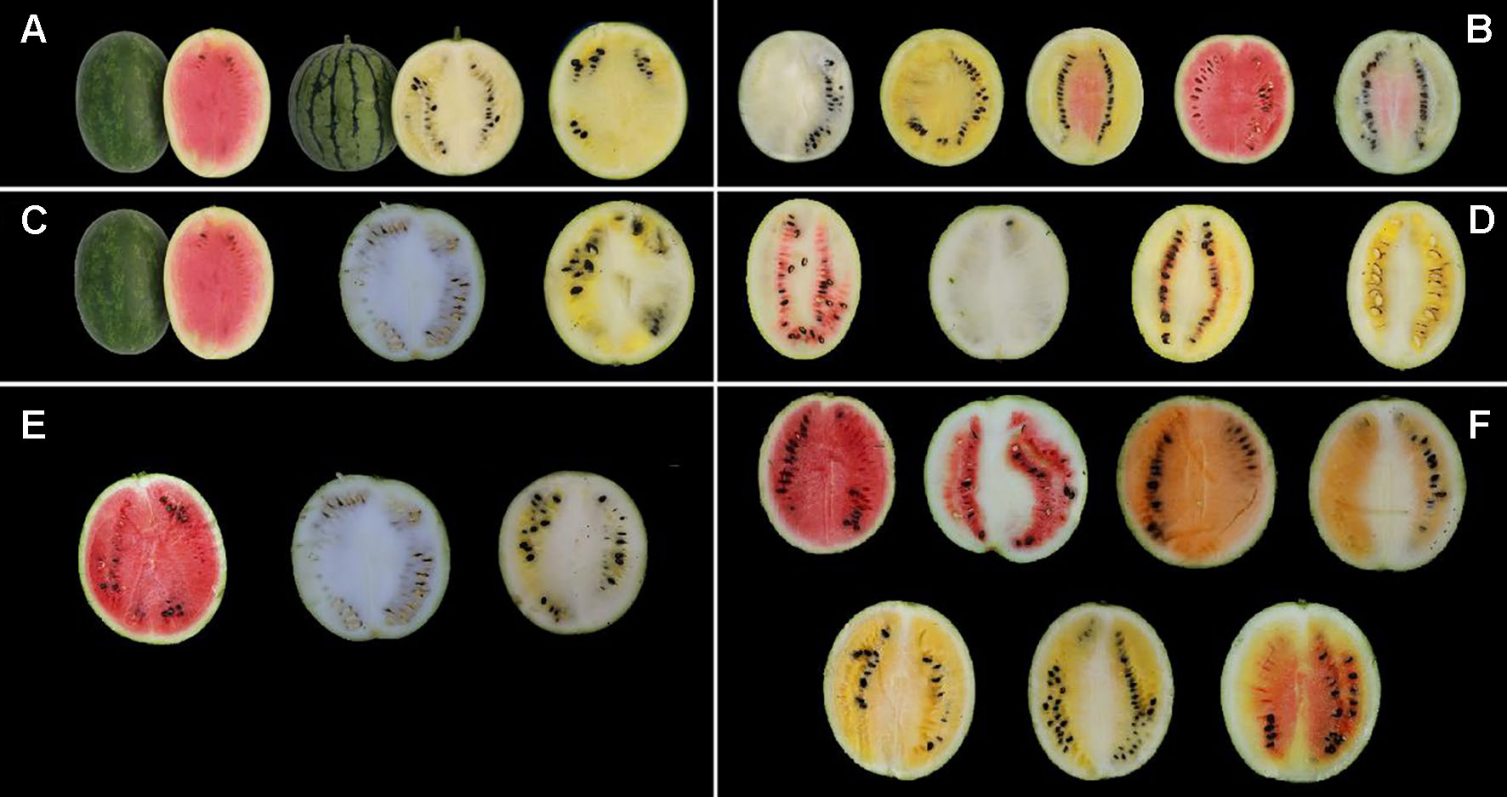
Phenotype pictures of the parental lines, F_1_ generation, F_2_ populations, and BC_1_P_1_ population derived from the four parental materials. **(A)** From left to right, LSW-177, COS, and F_1_ generation of LSW-177 × COS. **(B)** F_2_ generation of LSW-177 × COS segregated into different kinds of flesh color. **(C)** From left to right, LSW-177, PI 186490, and F_1_ generation of LSW-177 × PI 186490. **(D)** F_2_ generation of LSW-177 × PI 186490 segregated into different kinds of flesh color. **(E)** From left to right, garden female, PI 186490, and F_1_ generation of garden female × PI 186490. **(F)** BC_1_P_1_ generation of garden female × PI 186490 segregated into different kinds of flesh color.

A panel of 81 watermelon accessions (27 accessions were collected from the Germplasm Resources Information Network, US Department of Agriculture, Agricultural Research Service, and others were maintained in the Laboratory of Molecular Genetic Breeding in Melon and Watermelon, Horticulture College of Northeast Agricultural University, China), including white (14 accessions), yellowish (19 accessions), pink (6 accessions), and red (42 accessions) flesh color developed from the natural population, was used for flesh color MAS system construction and verification. All the plants were grown in the greenhouse on the experimental farm of XiangFang and XiangYang Experiment Agricultural Station of Northeast Agricultural University, Harbin (44°04′N, E125°42′), China, during the summer of 2014, 2015, and 2017. The flesh colors of the parental materials and the genetic populations are shown in **Figure 1**.

### Lycopene Measurement and Flesh Color Evaluation

All the plants were artificially pollinated with an identification tag showing the date of pollination, and the fully mature fruits were harvested between 35 and 50 days after pollination. The harvested fruits were cut longitudinally, photographed for reassessment, and categorized into different flesh groups by visual observation. Flesh color data were scored as 3 for red, 2 for canary yellow, and 1 for pale yellow in Pop. 1, whereas 1 was used for red and 0 for nonred in Pop. 2 and Pop. 3. Pulp samples were mixed equally with five parts taken from the central tissue and four other points around the edge of the mature fruits. The samples were maintained at −80°C for high-performance liquid chromatography (HPLC) analysis. A Waters PDA detector 2535 and Agilent LC ZORBAX SB-C18 column (4.6 × 250 mm, 5 µm) were used for lycopene content analysis. Lycopene authentic standard (Sigma, USA) was dissolved with dichloromethane to obtain a 5 μg/mL concentration solution. The retention time was referenced to identify lycopene with the UV-V spectra condition of 472 nm. Data were analyzed by BREEZE software (Waters), and the lycopene content was quantified by utilizing authentic standard curves and determined as µg/g flesh weight. The method and protocol of lycopene extraction and measurement were according to Yuan et al. (2012) and Liu et al. (2015).

### Statistical Analysis

The mean values, standard deviations, trait distributions, and χ^2^ test analysis, and other statistical analyses were evaluated with SPSS 19.0 software (SPSS Inc., United States)

### DNA Preparation

Young leaf tissues of 15 plants from each parental material and F_1_ generation were used for DNA extraction. The DNA of Pop. 1, Pop. 2, Pop. 3, fine mapping population plants, and the 81 accessions was extracted individually with a modified CTAB (hexadecyl trimethyl ammonium bromide) method as reported previously (Luan et al., 2008).

### Marker Development, PCR, and Gel Electrophoresis

#### Caps Markers

CAPS markers were developed based on resequencing of the four parental watermelon lines with the Illumina HiSeq 2000 high-throughput sequencing platform. In total, 10 Gb of data were obtained from each material, covering the watermelon genome at more than 20×. The resequencing data analysis and marker development were performed according to Liu et al. (2015).

Twenty-two restriction endonucleases (*Eco*RI, *Bsa*HI, *Hind*III, *Mbo*II, *Pst*I, *Sca*I, *Bam*HI, *Mlu*I, *Alu*I, *Dra*I, *Pvu*I, *Kpn*I, *Ras*I, *Hind*II, *Taq*I, *Msp*I, *Sac*I, *Bcl*I, *Xho*I, *Ned*I, *Sac*I, and *Mbo*I) (Thermo Scientific, Massachusetts, United States) were used for marker development. CAPS loci were selected on each chromosome for primer design, and amplicons derived from the DNA of both parents and the F_1_ generation were digested with restriction endonucleases. The PCR mixture and conditions for the CAPS markers were reported previously (Liu et al., 2015). The reaction mixture and conditions for the enzyme digestions were determined from the manual of each restriction endonuclease (Thermo Scientific). Finally, 1% agarose gel electrophoresis was used to examine the enzyme-digested products.

### SSR and Indel Markers

In total, 449 pairs of watermelon SSR markers (including 23 pairs of core watermelon SSR markers, Zhang et al., 2011) and 556 pairs of melon SSR markers were used for polymorphism selection. All SSR markers were derived from available publications (Danin-Poleg et al., 2001; Fazio et al., 2002; Silberstein et al., 2003; Yi et al., 2003; Gonzalo et al., 2005; Joobeur et al., 2006; Zalapa et al., 2007; Fernandez-Silva et al., 2008). Eighteen pairs of SSR and indel markers (including 17 SSR markers and 5 indel markers) used for mapping yellow fruit flesh traits in cucumber (Lu et al., 2015) were also chosen in this research. The PCR mixtures for SSR and indel amplification were the same as those used for the CAPS markers, and the conditions were according to Lu et al. (2015). The PCR products were analyzed with 6% denaturing polyacrylamide gel electrophoresis and visualized by silver staining.

### Map Construction and Secondary and Fine Mapping of the Candidate Region

Cleaved amplified polymorphism sequence (CAPS) markers were developed both in the preliminary mapping region and throughout the whole genome for fine mapping. Twenty-six pairs of new CAPS primers were designed in the preliminary mapping region of *LCYB 4.1*, among which eight pairs showed polymorphisms between LSW-177 and COS. Some CAPS markers were also designed based on a sequence alignment with zeaxanthin epoxidase, which has been reported to show differential expression among red-, yellow-, and white- fleshed watermelon during growth period (Nakkanong et al., 2012 and Lv et al., 2015). At the same time, the SSR marker *SSR17292* (Lu et al., 2015) for identifying yellow flesh in cucumber performed polymorphism between LSW-177 and COS was used. Subsequently 1,202 extended population (from the same source of Pop. 1) were used for fine mapping. And in the candidate region, 15 pairs of CAPS were developed in the fine mapping interval, and 4 pairs of polymorphic markers were included.

Genetic linkage map construction was performed using the IciMapping V3.3 software (Institute of Crop Science Chinese Academy of Agricultural Sciences, Beijing, China; Meng et al., 2015), and all the markers were grouped at a minimum logrithm of odd (LOD) score of 6.0. The software package Map Chart 2.1 (Plant Research International, Wageningen, the Netherlands; Voorrips, 2002) was used to graphically represent the linkage groups in the map. QTL analysis was also performed with IciMapping V3.3 (Meng et al., 2015), and QTLs with a LOD score ≥5.0 were considered as the available loci for detection. The fine mapping region was narrowed with the recombinant plants.

### Sequence Annotation and Gene Prediction in the Genomic Region Harboring the Target Gene

The genome data of the four parental materials and another 20 watermelon accessions that have been published (Guo et al., 2012) were used for sequence alignment with the watermelon reference genome sequence (97103) from the Cucurbit Genomics Database (Zheng et al., 2018). Using the open reading frame (ORF) and Basic Local Alignment Search Tool (BLAST; Altschul et al., 1900) analysis to detect the candidate genes in the mapping region, only sequences that matched with an identity of more than 95% were retained. The sequence of the coding sequence (CDS) and candidate genes were aligned to detect the splice sites using the Splign tool (Kapustin et al., 2008). Candidate gene sequence alignment and amino acid variation were performed using DNAMAN 6.0 (Lynnon Biosoft, USA) software.

### Validation of Watermelon Flesh Color Markers for MAS Breeding

To develop practical molecular markers for MAS breeding, the markers cosegregating with the lycopene content and flesh color locus were validated using Pop. 3 and 81 watermelon accessions to investigate the correlation between the genotype and phenotype data.

### Construction of Phylogenetic Tree

In the Cucurbitaceae genome database, the *LCYB* and *LCYE* homologous genes of different Cucurbitaceae crops were searched, with the addition of an *LCYB* gene from tomato, and then the encoded amino acid sequences were obtained. The amino acid sequences were compared by multiple sequence alignment using trimAI software (Capella-Gutierrez et al., 2009), and then MEGA (Kumar et al., 2018) was applied. A phylogenetic tree was established with the UPGMA function in MEGA X (Kumar et al., 2018) software.

### Transcript Expression Level of Candidate Genes in Different Watermelon Varieties

In order to explore the expression level of candidate genes in different flesh color watermelons, species 97103, PI 1296341, COS, and LSW-177 were selected for the goal varieties from the database http://cucurbitgenomics.org/ (the RNA-sseq data of COS and LSW-177 were the target varieties in this research). Based on the transcript expression (RPKM) of candidate genes *Cla005011* and *Cla005012* in the above four varieties, we aggregated their data and produced a trend graph using software GraphPad 8.0. We analyzed the expression levels of the two candidate genes at different mature stages of different flesh color watermelons.

## Results

### Phenotypic Segregation Analysis of Flesh Color and Lycopene Content

The flesh color of the F_1_ generation was canary yellow in Pop. 1, close to pale yellow, suggesting pale yellow with an incomplete dominance over red. Five categories of flesh color, red (87 plants), pale yellow (48 plants), canary yellow (173 plants), and two irregular color patterns consisting of red mixed with pale and canary yellow or red in mixed patterns in the heart and placental tissues of the fruit (18 and 26 plants, respectively), were found in the segregating population. Most of the mixed pale and canary yellow fruits had flesh color >50% canary or pale yellow by cross-sectional area, so the two mixed-color plants could be classified as canary yellow and pale yellow. According to these classification criteria, 199 (173 + 26), 66 (48 + 18), and 87 plants were judged to have canary yellow, pale yellow and red flesh color in Pop. 1, fitting a genetic segregation ratio of 9:3:4 (χ^2^  =  0.02 and 1.12, *P*  =  0.99 and 0.57 for the year of 2013 and 2014, respectively), which indicated that flesh color was affected by two major genes. Canary yellow and pale yellow could also be classified into red and nonred groups by visual observation. The segregation of these two groups yielded a ratio that did not differ significantly from a 3:1 ratio (nonred group: red group  =  265:87, *P* < 0.05) by statistical analysis. These results indicated that a single major recessive gene determined red and nonred color in watermelon based on the genetic background of Pop. 1.

**Figure 2:**
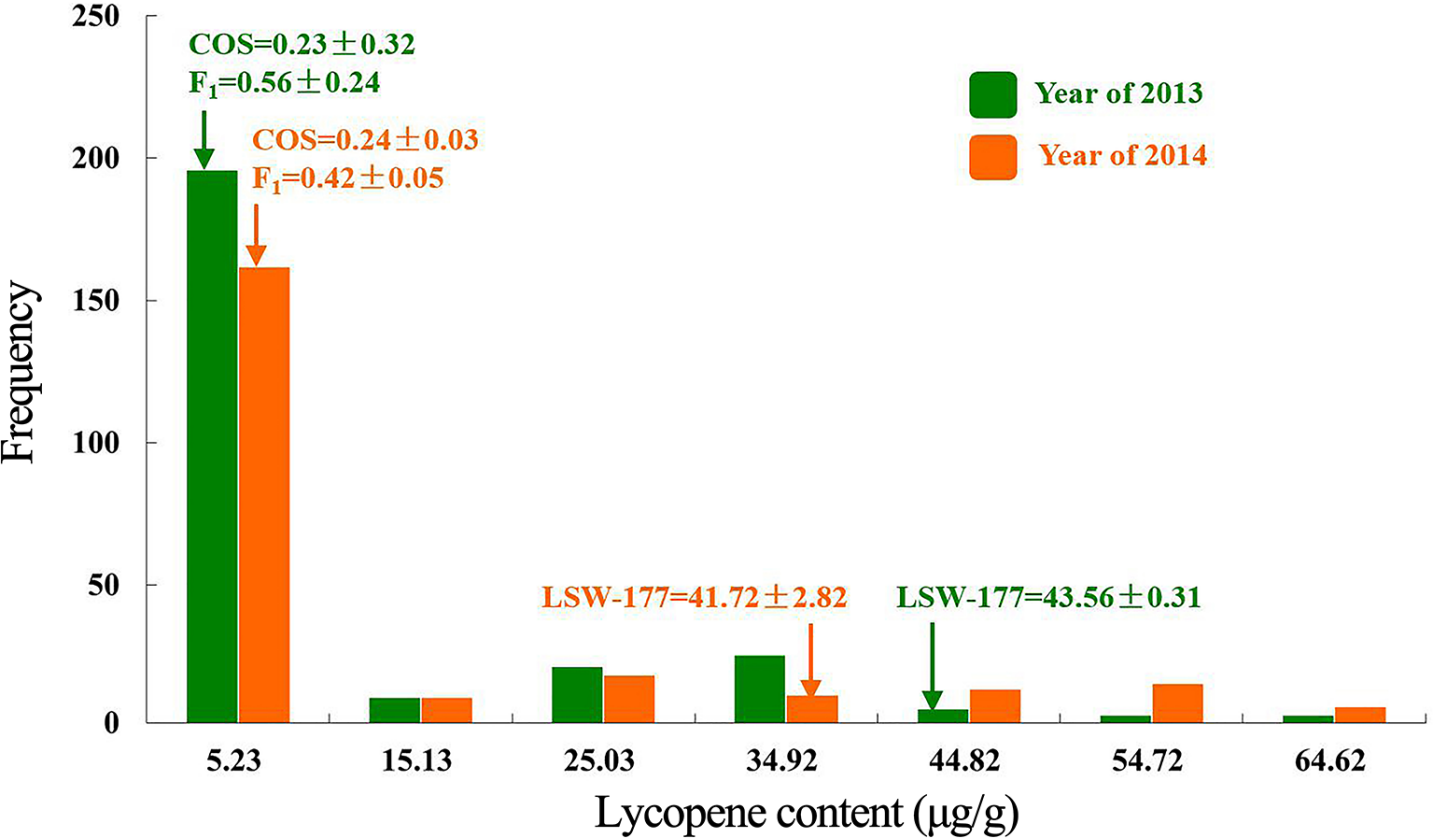
Segregation analysis of lycopene content in the F_2_ population from a cross between LSW-177 and COS parental strains in the year of 2013 and 2014.

High-performance liquid chromatography analysis of lycopene contents in mature fruit showed that LSW-177 had a high lycopene content with an average value of 41.72 ± 2.82 µg/g, much higher than 0.24 ± 0.03 and 0.42 ± 0.05 µg/g measured in COS and the F_1_ generation, respectively. Comparing the lycopene content and flesh color data, we observed that the plants showed a red flesh color when the lycopene content was higher than 13.57 µg/g. Pop. 1 could also be divided into two groups (high- and low-lycopene groups) at this threshold value. The genetic ratio of the high-lycopene (87 plants) to low-lycopene (265 plants) groups in the F_2_ progeny adequately fitted a 1:3 (χ^2^  =  0.015, *P*  =  0.902) ratio, demonstrating that one major gene affects lycopene accumulation. We also compared these results with our previously published data (Liu et al., 2015) from 2013, when we collected lycopene content data in an F_2_ generation (234 plants) with the same parental materials. The F_2_ generations in both years showed the same genetic ratio for flesh color and similarly divergent trends in lycopene content. The results of the χ^2^ goodness-of-fit test of the segregation ratios and the lycopene content separation analysis in the F_2_ populations derived from LSW-177 and COS are shown in **Table 1** and **Figure 2**.

**Table 1:** Flesh color separation proportion of the parental materials and F_2_ population.

Generation	Canary yellow	Pale yellow	Red	Expected ratio	χ^2^	*P*
P_1_	0	0	30	0:0:1	0	1
P_2_	0	25	0	0:1:0	0	1
F_1_	30	0	0	1:0:0	0	1
F_2_ (2014)	199	66	87	9:3:4	0.02	0.99
F_2_ (2013)	129	50	55	9:3:4	1.12	0.57

In Pop. 2, the flesh color of the F_1_ generation was canary yellow mixed with white. Four main flesh colors, red mixed with white, red mixed with white and yellow, canary yellow mixed with white, and white, emerged in the F_2_ generation. For the red and white mixed groups, most of the plants accumulated lycopene only around the seed region, while only two plants had fully red flesh color. Two groups were separated based on flesh color, one group with red or red mixed with white flesh and the other group with yellow, white, and mixed yellow and white flesh. The numbers of individuals in these two groups were 97 and 262, fitting a genetic segregation ratio of 1:3 (χ^2^  =  0.780, *P*  =  0.377).

In Pop. 3, the F_1_ plants had canary yellow mixed with white flesh. Approximately seven main flesh colors (red, red mixed with white, orange, orange mixed with white, canary yellow, canary yellow mixed with white and mixed canary yellow, orange and red) segregated in the BC_1_P_1_ generation. According to the classification standards of Pop. 2, two groups were also separated, one with red and red mixed with white, and the remaining fruits with other flesh colors. The genetic segregation of these two groups was 116:106, fitting a ratio of 1:1 (χ^2^  =  0.450, *P*  =  0.502). Considering that the F_1_ plants of Pop. 2 and Pop. 3 did not have white flesh color similar to that of PI 186490, the white flesh trait has incomplete dominance over red flesh in watermelon. The separate proportions of the three genetic populations are shown in **Table 2**.

**Table 2:** The separate proportion of red and nonred plants in the three genetic populations.

Population	Red	Nonred	Expected ratio	χ^2^	*P*
Pop. 1	87	265	1:3	0.015	0.902
Pop. 2	97	262	1:3	0.780	0.377
Pop. 3	116	106	1:1	0.450	0.502

### Secondary Mapping of the Lycopene Content and Red Flesh Color Loci Using Genome Resequencing Data

**Figure 3:**
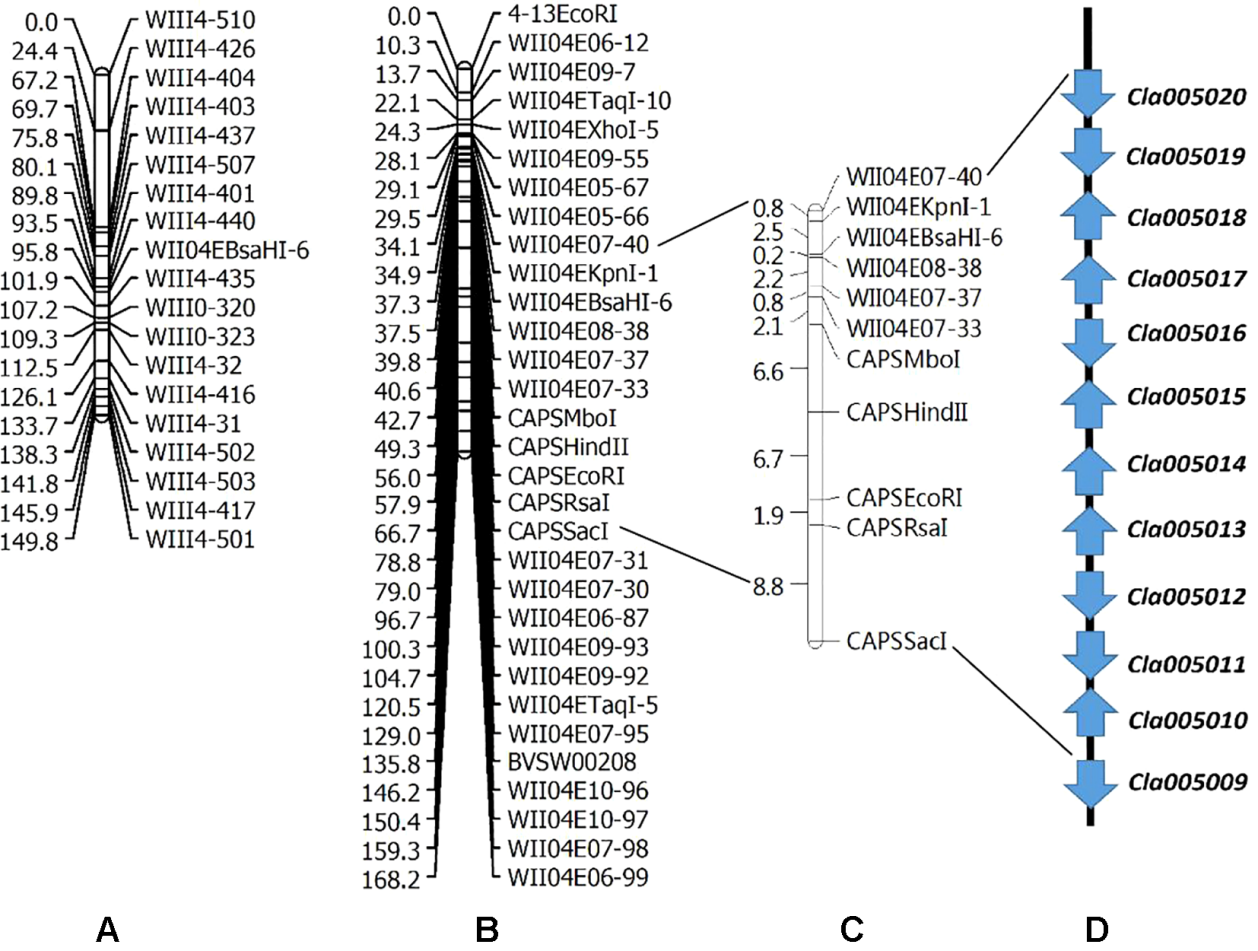
Genetic linkage groups of chromosome 4 for QTL detection and candidate genes in the effective region. **(A)** Genetic linkage group of chromosome 4 with the F_2_ generation (359 plants) derived from a cross between garden female and PI 186490. **(B)** Genetic linkage group of chromosome 4 with the F_2_ generation (352 plants) derived from a cross between LSW-177 and COS. **(C)** Linkage analysis of the CAPS markers in the QTL effective region. **(D)** Candidate genes in the QTL detected region.

Two linkage maps consisting of 311 markers (274 CAPS, 37 SSR) and 200 CAPS markers were constructed from Pop. 1 and Pop. 2 (**Figures S1** and **S2**). Eight new CAPS markers were developed in the initial mapping region for secondary mapping (**Figure 3**). The order of the CAPS marker locations did not tightly correspond to the reference genome. For Pop. 1, one major QTL that related to both the lycopene content and red flesh color traits shared the same candidate region on chromosome 4 between the newly developed CAPS markers *WII04E08-38* and *WII04EBsaHI-6*, 0.15 and 0.05 cM away from each marker, with high *R* ^2^ values (84.5% and 81.5%) and LOD scores of 86.3 and 91.21, respectively. Based on the genome resequencing data, the physical distance between *WII04E08-38* and *WII04EBsaHI-6* was 92,931 bp. The 11 CAPS markers (from *WII04E07-40* to *CAPSSacI* on chromosome 4; **Figure 3B**) confirmed the chromosome region, with 12 candidate genes cosegregating with flesh color in most individuals of Pop. 1. All the mixed-color plants were heterozygous, similar to the F_1_ generation.

Five candidate genes (*Cla005011* to *Cla005015*, which are presumed to encode proteins, **Table S1**) were detected in the 92,931-bp region (*WII04E08-38* to *WII04EBsaHI-6*) by consulting the Cucurbit Genomics Database (Zheng et al., 2018). Based on the results of ORF and BLAST (Altschul et al., 1900) analysis, the lycopene β-cyclase (*LCYB*) mRNA, LCYB-red allele, and CDS were located in this region with high sequence similarity to *Cla005011* (8,886,138 to 8,887,652), which indicated that although *SSR17292* related with yellow flesh color in cucumber (Lu et al., 2015) and also exhibited polymorphic between LSW-177 and COS, no QTL locus was detected. In Pop. 2, a major QTL (with an *R* ^2^ of 70.54%) between LSW-177 and PI 186490 was preliminarily located in the same region as in Pop. 1, with the flanking CAPS markers of *WIII4-440* (8,348,876) and *WIII4-435* (10,211,848) on chromosome 4. We also used LSW-177 and PI 186490 to determine the polymorphism status of the 11 CAPS markers that cosegregated with flesh color in Pop. 1, and four markers (*WII04EBsaHI-6*, *WII04E08-38*, *CAPSMboI*, and *CAPSHindII*) were found to be polymorphic. Based on the results of the linkage analysis, only *WII04EBsaHI-6* was assigned to the region between *WIII4-440* and *WIII4-435*, and it narrowed the effective QTL region with an *R* ^2^ value of 64.60%. *CAPSMboI* was polymorphic between LSW-177 and PI 186490, but in Pop. 2, all the individuals had the same alleles as LSW-177. The genotyping data showed that *WII04E08-38* and *CAPSHindII* had distorted segregation in Pop. 2, but these markers were distant from *WII04EBsaHI-6* and the target region. This result may reflect the fact that PI 186490 belongs to *C. lanatus* subsp. *mucosospermus*, which is another subspecies of watermelon. Considering the genome divergence of different watermelons, separate reference genomes are needed. For the CAPS marker *WII04EBsaHI-6*, all plants having red mixed with white flesh color showed the same electrophoretic bands as LSW-177, and the individuals with mixed flesh colors were heterozygous, similar to the F_1_ generation.

### Fine Mapping of the Red Flesh Color Loci

**Figure 4:**
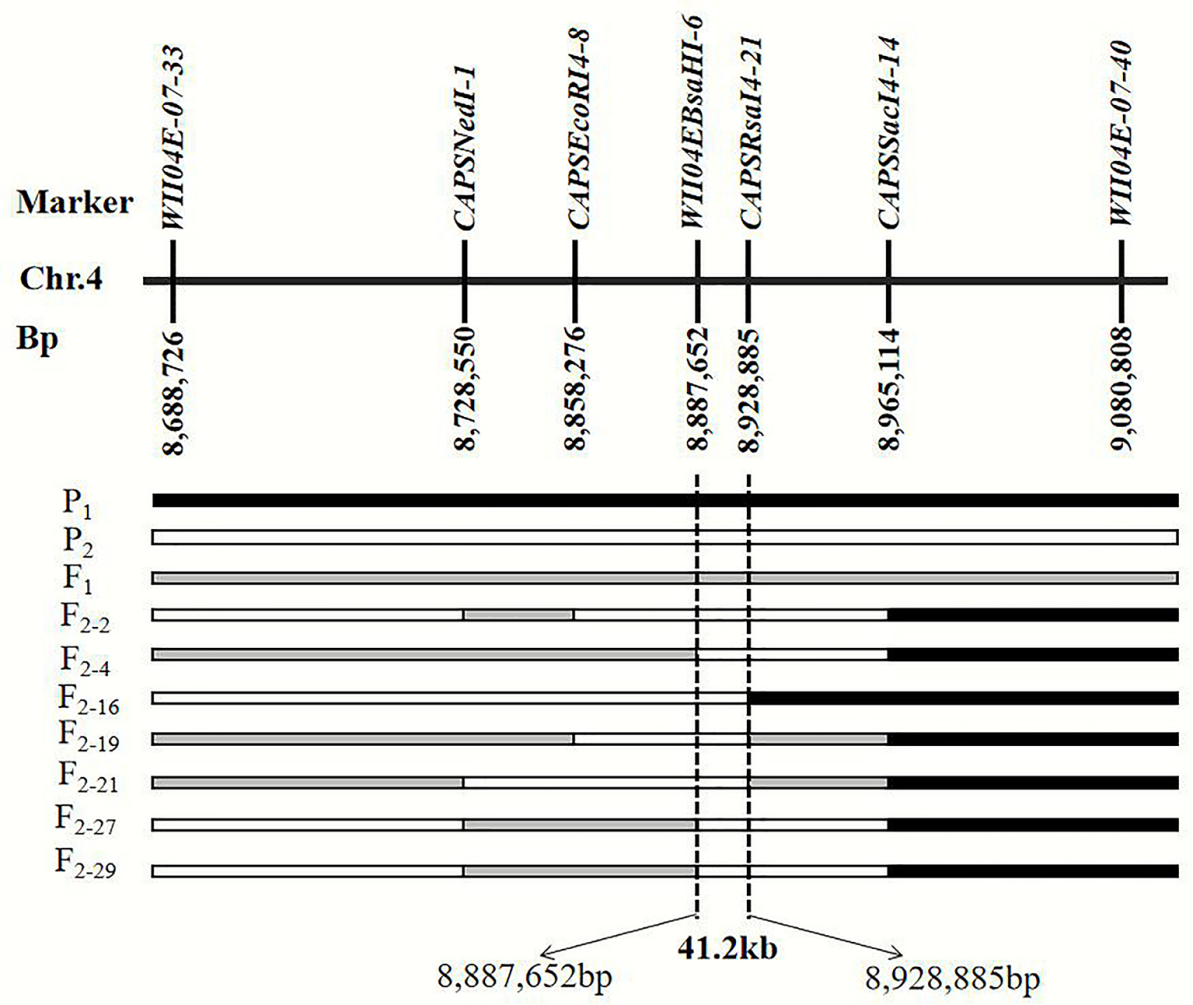
Fine mapping of the genes that control the color of watermelon red flesh through recombinant recessive individuals. P_1_ black frame represents the parent material LSW-177, P_2_ white frame represents the parent material PI 186490, F_1_ was gray frame. F_2-2_, F_2-4_, F_2-16_, F_2-19_, F_2-21_, F_2-21_, F_2-27_, and F_2-29_ were recessive recombinant individuals, respectively.

By filtering the recombinant plants using CAPS markers *WII04E08-33* and *WII04E08-40* in the F_2_ population, which was derived from the enlarged population of Pop. 1 containing 1,202 individuals, 28 recombinant individuals were found. Among these, 4 dominant homozygous individuals, 6 recessive homozygous individuals, and 18 recombinant individuals were identified. Four CAPS markers (*CAPSNedI-1*, *CAPSEcoRI4-8*, *CAPSRsaI4-21*, *CAPSSacI4-14*) and one known marker, *WII04EBsaHI-6*, were developed between the *WII04E08-33* and *WII04E08-40* markers. The target region between markers *WII04EBsaHI-6* and *CAPSRsaI4-21* was ultimately acquired by using the above markers to fine map the six recessive homozygous individuals (red flesh), and the physical distance was 41.2 kb (**Figure 4**). Using the Cucurbit Genomics Database (Zheng et al., 2018), two candidate genes, *Cla005011* and *Cla005012*, were found in the fine mapping region between the positions from 8,886,138 to 8,926,873.

**Figure 5:**
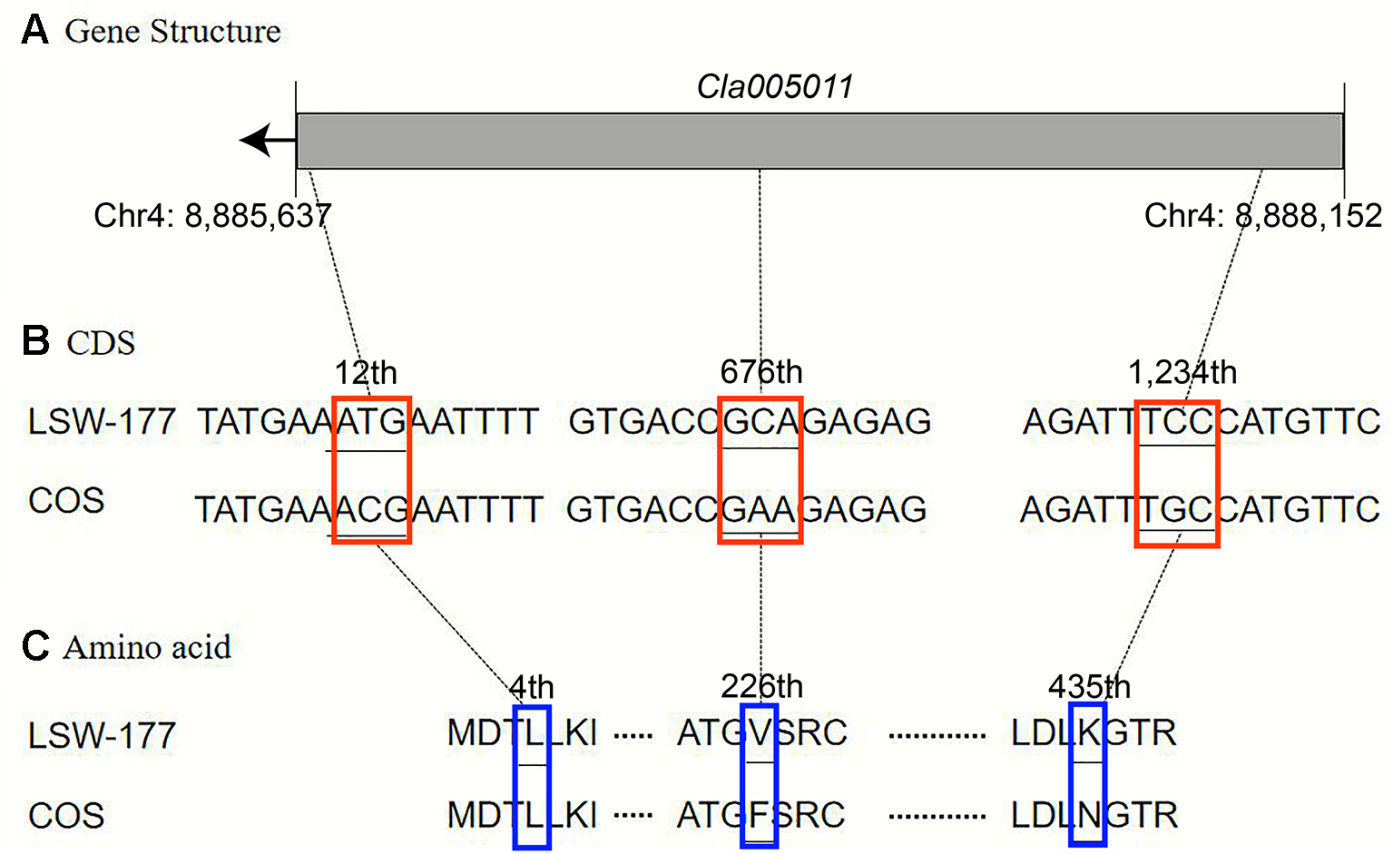
Comparison analysis of the DNA sequence and amino acid sequence of the *Cla005011* between LSW-177 and COS. A 3-bp mutation resulted in two amino acid mutations: **(A)** gene structure of *Cla005011*, including only one exon; **(B)** coding sequence analysis of three SNP mutations; **(C)** two amino acids mutation due to three SNP mutations. The black boxes indicate the exons.

**Figure 6:**
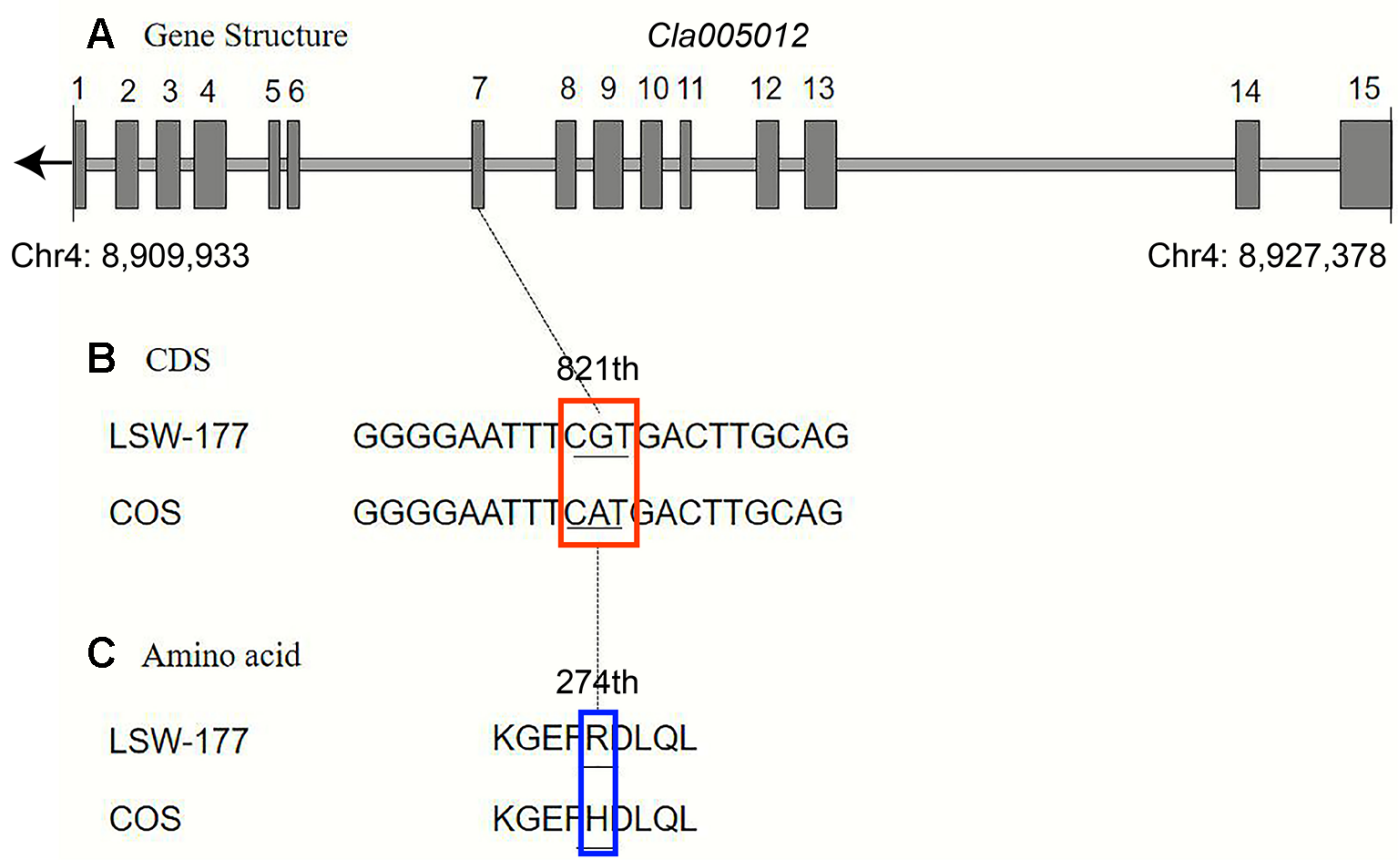
Comparison analysis of the DNA sequence and amino acid sequence of the *Cla005012* between LSW-177 and COS. A 1-bp mutation resulted in one amino acid mutation: **(A)** gene structure of *Cla005012*, including 15 exons; **(B)** coding sequence analysis of one SNP mutations; **(C)** one amino acid mutation due to one SNP mutation. The black boxes indicate the exons.

Upon comparison and analysis of the candidate gene sequences between the two parents with the resequencing data, three SNP mutations were found in the exon of the candidate gene (*Cla005011*), among which two SNPs led to two amino acid changes (mutation of the 676th base G/C to T/A resulted in the 226th amino acid changing from valine [V] to phenylalanine [F], and mutation of the 1,234th base G/C to C/G resulted in the 435th amino acid changing from lysine [K] to asparagine [N]). No amino acid changes were detected with the third SNP mutation (although the 12th base was mutated from A/T to G/C, it did not cause amino acid changes; **Figure 5**). A single-nucleotide mutation within the exon of the candidate gene (*Cla005012*) led to one amino acid change (the 821th base had a G/C to A/T mutation, resulting in the 274th amino acid changing from arginine [R] to histidine [H]; **Figure 6**). Furthermore, according to fine mapping and combination of the results of CDS blasting with the gene *LCYB*, *Cla005011* was speculated to be on the same locus as *LCYB* in watermelon.

### Genetic Effect of LCYB4.1 on Lycopene Content in Pop. 1

According to the genotyping data, the 11 CAPS markers perfectly cosegregated with flesh color and lycopene content in Pop. 1. To further validate the results of the QTL mapping, we also tested the relationships between lycopene content and the CAPS marker allele pairs (*WII04EBsaHI-6* and *WII04E08-38*) for all individuals in Pop. 1 (**Table S2**). For the CAPS marker *WII04EBsaHI-6*, the average content of lycopene in plants with homozygous LSW-177 alleles was 37.38 ± 13.24 µg·g^−1^, while the plants with homozygous COS alleles had a value of 0.23 ± 0.06 µg·g^−1^. Heterozygous individuals accumulated an average content of 2.62 ± 8.10 µg·g^−1^. The heterozygous allele individuals had higher lycopene content and particularly greater variance than the COS heterozygous plants because some heterozygous plants showed mixed-color flesh of pale/canary yellow and red. The same trend was also observed for *WII04E08-38*.

### Comparison of LCYB Sequences in Watermelon

The genome data of 4 parental materials and 20 other published watermelon accessions (Guo et al., 2012) were used to extract the sequence of the *LCYB* gene for sequence alignment and variation detection. The 24 watermelon samples exhibited four flesh colors: red, yellowish, green, and white. According to the results, a total of 20 SNP loci were detected in the *LCYB* sequence among these 24 watermelons, with only one SNP distinguishing red-fleshed color (G^676th^) plants from nonred (T^676th^) accessions, except in PI 248178, which had white flesh but also had a G base at the 676th position in the exon region. This SNP (G^676th^ to T^676th^) changed valine (V: red) into glycine (nonred). It was also the position of the restriction site of the CAPS marker *WII04EBsaHI-6* (**Figure S3**).

### Marker Utilization and MAS for Flesh Color in Watermelon

To verify the applicability of these markers in different genetic populations and watermelon accessions, the 11 CAPS markers in the effective region were used for genotyping in Pop. 3 and a watermelon panel. Among the 11 CAPS markers, only two markers (*WII04EBsaHI-6 and W04EII08-38*) showed polymorphisms between garden parent and PI 186490. In Pop. 3, two flesh colors (red and red mixed with white) appeared in individuals homozygous for the same alleles as garden parent, while plants with other flesh colors carried heterozygous alleles, similar to the F_1_ generation.

Considering the flesh color–specific markers and the different polymorphisms of white-fleshed plants, *WII04EBsaHI-6*, *W04EII08-38*, *WII04EKpnI-1*, and *WII04E07-40* were selected to construct a MAS approach for red, yellowish, and white flesh color in 81 watermelon accessions. Based on the genotyping results, the CAPS markers *WII04EBsaHI-6* and *W04EII08-38* cosegregated with red and pink flesh color in these accessions, except in two PI lines (PI 193963 and PI 601228, which had yellowish flesh but exhibited the same restriction enzyme fragments as the red and pink plants). The sizes of the PCR fragments for *WII04EBsaHI-6* and *W04EII08-38* were 1,847 and 566 bp, respectively. Plants with pink and red flesh colors showed enzyme-cut fragments of 1,182 and 665 bp for *WII04EBsaHI-6* and 566 bp for *W04EII08-38*, while the yellowish- and white- fleshed accessions showed fragments of 1,847 and 287 or 279 bp for these two markers (since the resolution ratio of the 1% agarose gel was 100 bp, the 287- and 279-bp products appeared as one band) (**Figure S4**).

For the CAPS markers *WII04EKpnI-1* and *WII04E07-40*, the PCR amplicons were 517 and 618 bp for all 81 accessions. In the yellowish group, each plant exhibited 389- or 128-bp bands for *WII04EKpnI-1* and 460- or 158-bp bands for *WII04E07-40*. The pink, red, and white plants showed 517- and 618-bp bands with these two markers. Three white-fleshed PI lines (PI 248774, PI 532666, and PI 254622) had the same results as the yellowish-fleshed plants. Some white-fleshed PI lines were heterozygous at these marker loci. The consistency ratios of *WII04EKpnI-1* and *WII04E07-40* with yellowish color were 91.4% and 92.6%, respectively (**Figure S5**).

To distinguish the three types of flesh color (red, yellowish, and white), the four markers were combined for further testing. Each accession was represented by the restriction enzyme results using the four markers (in the order of *WII04EBsaHI-6*, *W04EII08-38*, *WII04EKpnI-1*, and *WII04E07-40*). Plants with the marker restriction site were scored as 1, those without were scored as 0, and heterozygotes were recorded as h. With this method, each material could be given a marker code for flesh color. The results showed that the combination of these four markers could clearly distinguish the three flesh colors. According to the genotyping results (**Table S3**), all of the red and pink plants had the code 1, 0, 0, 0, whereas the code for the yellowish plants was 0, 1, 1, 1. For white-fleshed plants, the codes were not uniform, and heterozygous loci were present in some plants, but the code of each white-fleshed watermelon was quite different from those of the pink, red, and yellowish accessions. With these marker codes, we can easily distinguish the three types of flesh color in watermelon. The SSR marker *SSR17292* also showed different polymorphisms in the 81 watermelon accessions but did not cosegregate with flesh color.

### Expression Levels of Candidate Genes in Different Watermelon Varieties Based on Published RNA-Seq Data

**Figure 7:**
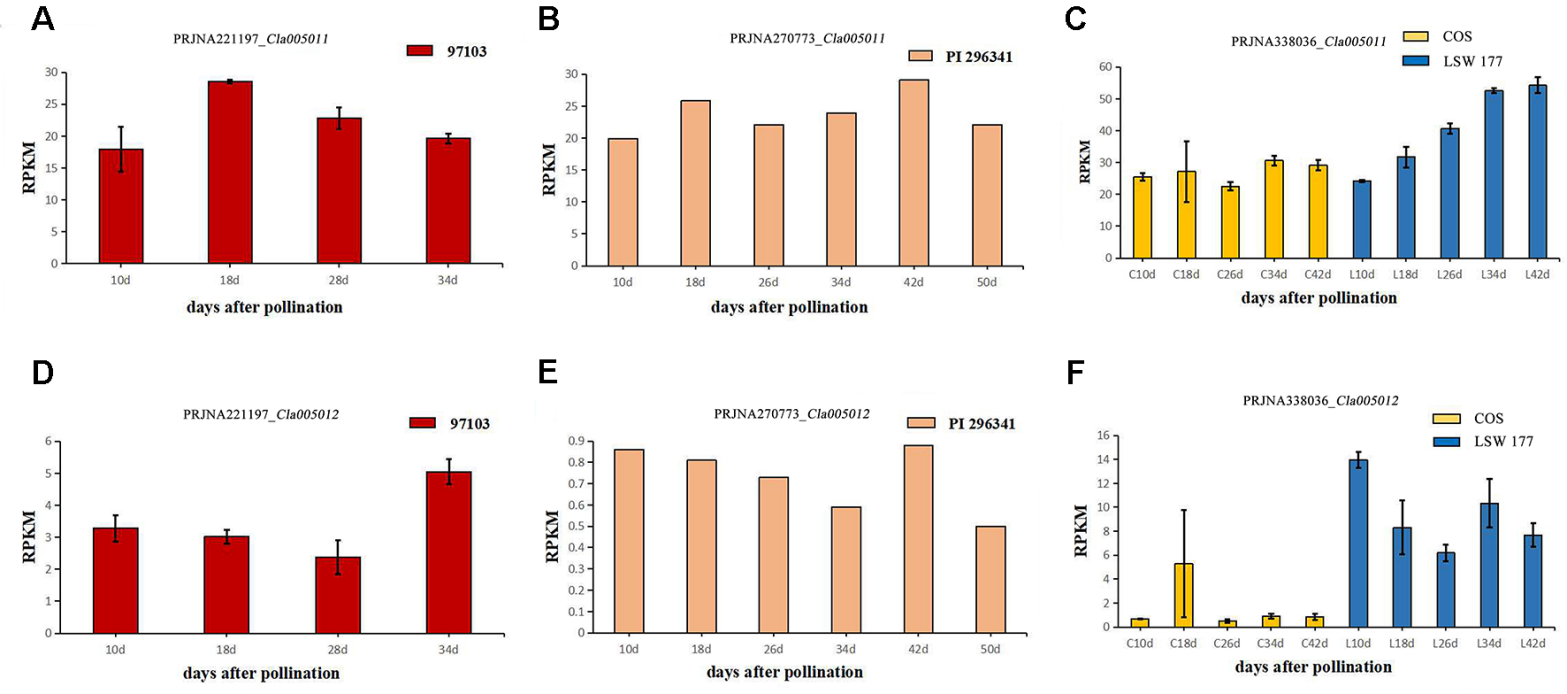
The expression levels of two candidate genes *Cla005011* and *Cla005012* in four different watermelon varieties (97103, LSW-177, COS, and PI 296341) at different maturity stages of the flesh. *Cla005011*: **(A)** BioProject: *PRJNA221197* (RPKM of 97103), sample DAP10, DAP18, DAP28, DAP34, DAP: days after pollination. **(B)** BioProject: *PRJNA270773* (RPKM of PI 296341), sample DAP10, DAP18, DAP26, DAP34, DAP42, DAP50. **(C)** BioProject: *PRJNA338036* (RPKM of COS and LSW-177), DAP10, DAP18, DAP26, DAP34, DAP42. *Cla005012*. **(D)** BioProject: *PRJNA221197* (RPKM of 97103), sample DAP10, DAP18, DAP28, DAP34. **(E)** BioProject: *PRJNA270773* (RPKM of PI 296341), sample DAP10, DAP18, DAP26, DAP34, DAP42, DAP50. **(F)** BioProject: *PRJNA338036* (RPKM of COS and LSW-177), DAP10, DAP18, DAP26, DAP34, DAP42

To explore the expression levels of select candidate genes in watermelon varieties with different flesh colors, four watermelon varieties were selected (according to the website http://cucurbitgenomics.org/): species 97103 (red flesh), PI 296341 (white flesh), COS (pale yellow flesh), and LSW-177 (red flesh). The transcriptional expression (RPKM) of two candidate genes, *Cla005011* and *Cla005012*, in the above species of watermelons was quantified at different periods of development. In all the accessions, COS, LSW-177, 97103 (red flesh), and PI 296341 (white flesh), the expression of *Cla005011* did not explain the lycopene accumulation difference between different watermelon accessions. *Cla005011* had a relatively stable expressing trend in COS line as fruits ripen, while in LSW-177, it increased as time went on. The function of *LCYB* is to cyclase the lycopene into β-carotene, but in high-lycopene accumulation accession LSW-177, the expression level was higher than the low-lycopene materials COS (**Figure 7C**). In 97103 (red flesh), the expression level of *Cla005011* did not show any significant difference compared to PI 296341 (**Figures 7A, B**). *Cla005012* was annotated as the kinesin-like protein that seemed to be not related with lycopene accumulation. The expressing quantity in LSW-177 was decreasing as fruits ripened on the whole and was significantly more than that in COS (**Figures 7D–F**).

### Phylogenetic Tree Analysis of the Lycopene Cyclase Gene Family

**Figure 8:**
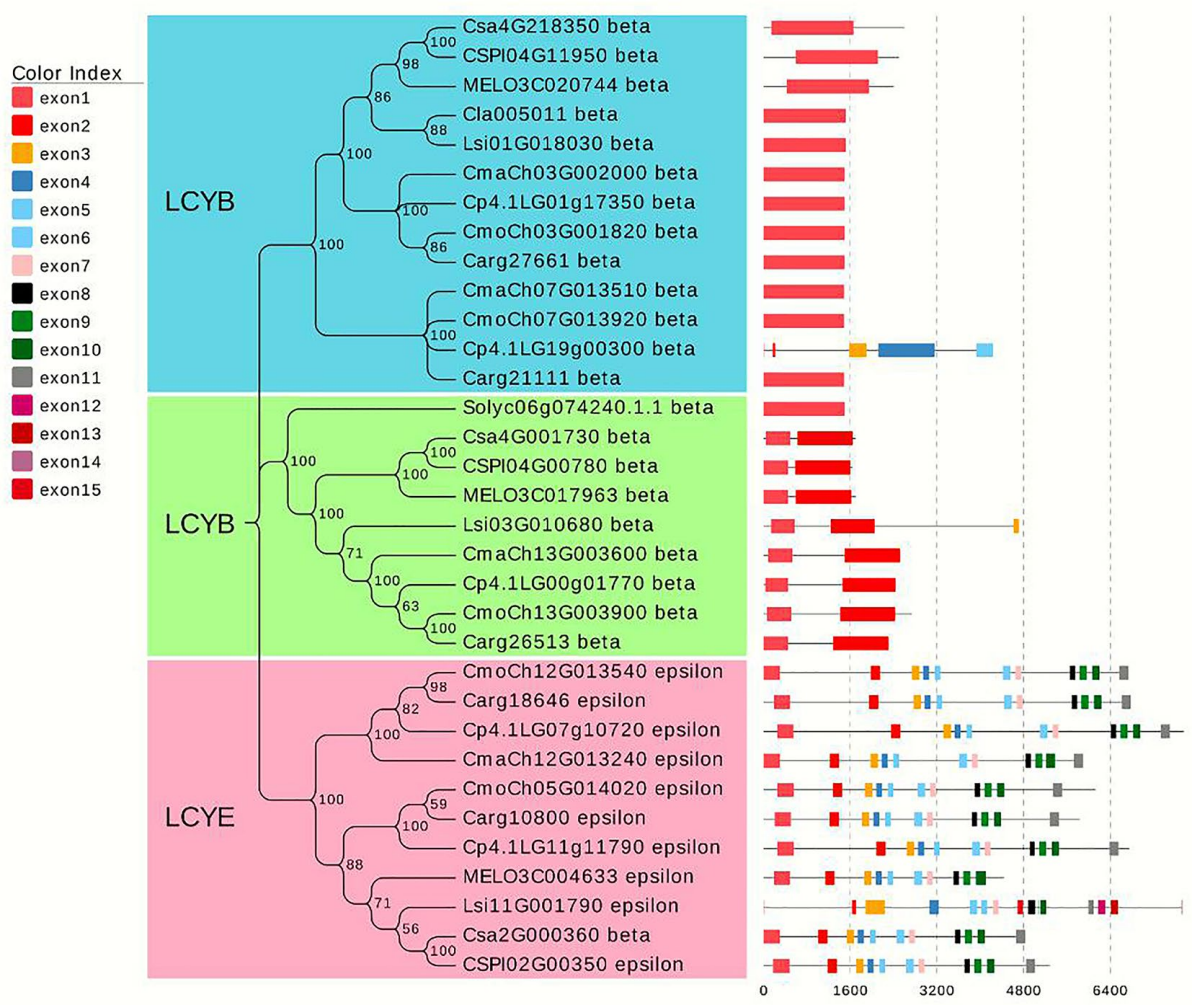
Phylogenetic tree analysis of lycopene homologous genes in different Cucurbitaceae crops and tomato. The phylogenetic tree was constructed by combining the lycopene genes of melon, watermelon, gourd, and pumpkin in Cucurbitaceae crops with one lycopene gene in tomato. A total of 33 homologous genes were divided into three clusters.

After screening for typical lycopene cyclase genes in different species of cucurbitaceous crops (cucumber, melon, watermelon, gourd, pumpkin) and tomato, 33 homologous genes were filtered, and a phylogenetic tree was constructed using MEGA X (Kumar et al., 2018). The results showed that all genes were divided into three categories, which were defined as lycopene β-cyclase (*LCYB*) and lycopene epsilon-cyclase (*LCYE*). *LCYB* clusters I and II contained 13 and 9 genes, respectively. Additionally, the number of exons was relatively consistent and conservative. Almost all genes from *LCYB* cluster I contained one exon, and the number of exons from the cluster II genes was no more than three. The remaining 11 genes from cluster III encoded *LYCE*, which regulates lycopene to generate the synthesis of α-carotenoids, which in turn produces lutein. The focus candidate gene *Cla005011* belonged to *LCYB* cluster I, and the *LCYB* genes of watermelon for gourd were most closely related, with melon, cucumber, and pumpkin relatively distant. Tomato species with higher levels of lycopene accumulation were used in the process of constructing the phylogenetic tree, and the *LCYB* genes of watermelon were further related to that of tomato. The phylogenetic tree analysis demonstrates that the candidate gene identified from the fine mapping results participated in regulating red flesh formation through the process of carotenoid production (**Figure 8**).

## Discussion

### Inheritance of Flesh Color and Lycopene Content

Red flesh color and lycopene content are major influences on watermelon quality and consumption. In previous research, the red flesh color trait in watermelon was found to be controlled by a single recessive gene (Henderson et al., 1998, Bang et al., 2010). To analyze the genetic inheritance patterns of red flesh color in watermelon, two F_2_ populations and one BC population were tested in our research. The segregation of these three populations further verified that one major recessive gene controlled red flesh color and lycopene content. The segregation of flesh color in Pop. 1 was 9:3:4 in both 2013 (Liu et al., 2015) and 2014, and we propose that the gene for pale yellow (*Py*) flesh in watermelon is epistatic to the canary yellow–related gene based on the appearance of pale yellow flesh color in Pop. 1. The homozygous recessive allele of the *py* gene could inhibit the formation of canary yellow, yielding a pale yellow flesh color. The results from the HPLC data of Pop. 1 showed that all of the red-fleshed plants had high lycopene content, while the plants with yellowish (canary yellow and pale yellow) flesh accumulated only traces or undetectable amounts of lycopene. The skewed distribution of lycopene content in Pop. 1 implied that a major effect gene controlled this trait.

Both LSW-177 and garden parent have red flesh color in their mature fruit, but their segregation was quite different when they were crossed with the white-fleshed PI 186490. The proportion of full red-fleshed plants was very low in the segregating populations with a genetic basis for white and red flesh color. Only two full red-fleshed plants were detected in Pop. 2, and in Pop. 3, there were approximately 22 full red fleshed individuals out of 116 plants. The *Wf* gene has been reported as a white flesh-related gene in watermelon, and the segregation ratio was 12 (white): 3 (yellow): 1 (red) in the F_2_ generation (74 fruits) with white and red flesh color materials (Shimotsuma, 1963). In the research of Zhang and colleagues, watermelon lines 97103 (red-fleshed) and PI 296341-FR (light yellowish green flesh color, which is regarded as a white-fleshed parental line) were used to form F_2_ and F_9_-RIL populations. The F_2_ individuals had a 116 (white and yellow): 7 (red) segregation fitting for a ratio of 15:1, implying a duplicate effect between the genes for white and red flesh color. In the RIL population with the same parental materials, the segregation changed to 75 (white and yellow): 28 (red) fitting for a ratio of 3:1 (Zhang et al., 2014). Another *Wf* gene investigation was reported by Gusmini and Wehner (2006), but in this study, COS, with pale yellow flesh color, was used as white-fleshed material to cross with a canary yellow flesh line, NC-517. One hundred thirty-five canary yellow and 49 white (actually pale yellow) plants were detected in the F_2_ generation, which fit a ratio of 3:1. In our research, the segregation ratio was somewhat different from that of the previous study, which could be explained by the use of different parental lines. PI 186490, with a pure white flesh color, was different from COS and PI 296341-FR. According to the genotyping data with the gene marker *WII04EBsaHI-6* in Pop. 2 and Pop. 3, plants with a red or red-mixed white flesh color had the same alleles as those of the red parental materials and other red accessions. The ratio of red alleles and nonred alleles was 1:3 and 1:1 for Pop. 2 and Pop. 3, respectively. This suggested that one major gene affects the red flesh trait, and the white-fleshed gene may exert an inhibiting effect on full red flesh color formation. The white flesh color trait was incomplete dominant to the red flesh trait based on the fact that the two F_1_ generations of Pop. 2 and Pop. 3 were yellowish mixed with white, not full white. The white-fleshed trait would be more complicated than pale yellow and red, which still needs further investigation.

Orange-fleshed plants were segregated in Pop. 3, while none were detected in Pop. 2, suggesting that the pigment compositions in LSW-177 and garden parent were quite different from each other; some carotenoid-containing orange flesh or a mixture of red and yellowish pigments accumulated in the mature flesh of garden parent.

### The Locus Related to Red Flesh Color and the Lycopene Content Trait

The carotenoid biosynthesis pathway in plants has been thoroughly investigated (Cazzonelli and Pogson, 2010), but little research was focused on QTL analysis of lycopene accumulation in watermelon. Red tomato is also a high-lycopene-content plant, which reportedly undergoes pigment development similar to that of watermelon (Grassi et al., 2013); however, the two fruits are still suggested to have different regulatory systems in their carotenoid biosynthesis pathways. The genes in the carotenoid biosynthesis pathway perform through different action modes to regulate carotenoid accumulation and flesh color formation. Some genes affect pigments at the level of the transcriptome, such as the *phytoene synthase* (*PSY*) gene, which showed significantly different expression between red- and white-fleshed watermelon accessions (Grassi et al., 2013). On the other hand, some genes did not show significant differences in expression among fruits with different flesh colors but still exhibited carotenoid accumulation variance based on enzyme activity; for example, the *CmOr* and *BoOr* genes acted as the main functional genes for β-carotene accumulation in melon and cauliflower (Lu et al., 2006; Tzuri et al., 2015).

By performing stepwise increases in mapping population sizes, marker numbers, and multiple genetic populations, a major effective QTL and candidate gene related to lycopene content and red flesh color was refined in a narrow region of chromosome 4. The two trait-related QTLs shared the same region, and the red flesh color gave a high correlation with the lycopene content in the F_2_ generation for Pop. 1. Based on the preliminary mapping information, new CAPS markers were developed to narrow down the region from 392,077 bp to 41,233 bp. To verify the stability of the QTL we identified, Pop. 2 and Pop. 3 were also used in our research. The same QTL region and markers showed a high detection efficacy for red flesh color or lycopene content in the two populations through linkage analysis and MAS.

In this study, *Cla005011* seemed to be the best candidate gene for lycopene accumulation and red flesh color formation. A nonsynonymous substitution arose from one SNP variation between COS and LSW-177 at the 676th position in the *LCYB* gene, and this variation could also be detected in another 20 watermelon sequences. We also performed a transcriptome analysis of COS and LSW-177 at different time points throughout the whole growth period with the RNA of pulp, the expression level of *Cla005011* was not the main reason for red and nonred flesh color formation. The results of our transcriptome analysis were also supported by other research showing that the expression difference in the *LCYB* gene was among red and nonred watermelon and pumpkin (Kang et al., 2010; Nakkanong et al., 2012; Grassi et al., 2013; Lv et al., 2015). This might indicate that the accumulation of lycopene was not dependent on the expression of *LCYB*. It was also likely that the difference in protein levels or functionality may regulate the color of the fruit (Wang et al., 2016). This seemed contradictory when we combined the results of QTL mapping and transcriptome analysis. In liverwort, the results of functional identification also proved that the *LCYB* gene had a lycopene degradation capability to produce β-carotene at the enzyme activity level (Takemura et al., 2014). It is reasonable to speculate that *LCYB* may regulate lycopene metabolism through protein level. The nonsynonymous SNP locus in *Cla005011* in the 676th coding region (**Figure 5** and **Figure S3**) may be the key site causing the change in enzyme activity, which may still need further investigation. Although the red flesh color or lycopene content trait was recessive to the pale yellow and white flesh colors, some mixed color individuals still had lycopene accumulation in their mature fruits. This suggested the existence of some other loci acting as regulatory factors or functional genes in addition to the *LCYB* gene, which affected the lycopene accumulation in watermelon.

### MAS Technology for Flesh Color in Watermelon

Flanking markers with small mapping intervals have the potential to increase MAS efficacy by reducing errors during selection. Two kinds of assisted selection markers for red flesh color in watermelon were generated by Bang et al. (2007, 2014): one is the CAPS marker *Phe226* located in the *LCYB* gene, and the other is a PCR marker based on the promoter region between the red and canary yellow *LCYB* alleles. According to our results, the CAPS marker *WII04EBsaHI-6* and *Phe226* shared the same location, and we also implemented a forward genetics strategy to demonstrate the significance of *LCYB* in lycopene accumulation in watermelon (red to nonred). Restriction enzyme digestion of *W04EII08-38*, *WII04EKpnI-1*, and *WII04E07-40* (*Mlu*I, *Kpn*I, and *Mbo*II) was more cost-effective than that of *WII04EBsaHI-6* and *Phe226* (*BsaH*I). For other flesh colors, digestion was still weak according to a previous study. In our research, we developed two CAPS markers that cosegregated with yellowish flesh color both in the individuals of the genetic population and those in the natural panel. Two yellowish, three white, and one pink-fleshed watermelon accessions did not match with the MAS results, and the sequence comparison also showed that PI 248178 had a white flesh color but was encoded by the same *LCYB* sequence as that of the red accessions. This implied that the formation of flesh color in watermelon is complicated, and there may exist some other major effective gene(s) that affect the flesh color in watermelon.

The amount of pigment accumulation led to varying degrees of watermelon flesh color exhibited in one color system, such as pink to red and pale yellow to canary yellow. In our research, different shades of flesh color in one color system validate the same MAS results. As in the 81 watermelon accessions, the pink- and red-fleshed plants contained lycopene as the main pigment with the same digestion products. Based on these results, we speculated that the main effective genes could determine the formation of the red color system (pink gradually changing to red) and the yellow color system (from pale yellow to canary yellow). However, for each color system, other genes may regulate the amount of pigment accumulation to direct the formation of different shades of flesh color.

## Data Availability Statement

The raw data supporting the conclusions of this article will be made available by the authors, without undue reservation, to any qualified researcher.

## Author Contributions

CW, LS, and FL conceived and designed the experiments. AQ, XF, SL, and PG performed the field experiments. WC performed the data analysis and wrote the manuscript. AD offered the germplasm. All authors read and approved the final manuscript. It is worth noting that CW and AQ are co–first authors.

## Funding

This research was supported by the National Key Research and Development Program (2018YFD0100703). This work was also supported by the National Nature Science Foundation of China (31601775 and 31572144), the “Young Talent” Project of Northeast Agricultural University (17QC06), the project of China Postdoctoral Science Foundation (2017M611345), and the China Agriculture Research System (CARS-25).

## Conflict of Interest

The authors declare that the research was conducted in the absence of any commercial or financial relationships that could be construed as a potential conflict of interest.

## Supplementary Material

Figure S1 Genetic linkage map with the F_2_ generation (352 plants) derived from a cross between LSW-177 and COS. The shaded area on linkage group 4 indicates the location of QTLs associated with red flesh color traits (*LCYB4.1*). Chr: Chromosome

Figure S2 Genetic linkage map with the F_2_ generation (359 plants) derived from a cross between garden female and PI 186490. Chr: Chromosome The shaded area on linkage group 4 indicates the location of QTLs associated with red flesh color trait.

Figure S3 The sequence alignment of *LCYB* gene with 24 watermelon accessions.

Figure S4 Marker analysis for red, yellowish and white flesh color watermelon accessions using CAPS marker *WII04EBsaHI-6* and *WII04E08-38*. **Supplementary Figure S4a** was the genotyping results of CAPS markers *WII04EBsaHI-6*; figure S4b was the genotyping results of CAPS markers *WII04E08-38*. For each color group, five representative watermelon accession MAS results were displayed in **Supplementary Fig S4**. Lane 1, 7, 13, 19 and 25 is the D2000 plus DNA marker. From the top to the bottom was 5,000, 3,000, 2,000, 1,000, 750, 500, 250, 100 bp fragments, respectively. From lane 2 to 6 are five red flesh color watermelon accessions; from lane 8 to 12 are five yellowish flesh color watermelon accessions; from lane 14 to 18 are five pink flesh color watermelon accessions; from lane 20 to 24 are five white flesh color watermelon accessions.

Figure S5 Marker analysis for red, yellowish and white flesh color watermelon accessions using CAPS marker *WII04EKpnI-1* and *WII04E07-40*. **Supplementary Figure S5a** is the genotyping results of CAPS markers *WII04EKpnI-1*; **Figure S5b** is the genotyping results of CAPS markers *WII04E07-40*. For each color group, five representative watermelon accessions MAS results are displayed in **Figure S5**. Lane 1, 7, 13, 19 and 25 are the D2000 plus DNA marker. From the top to the bottom are 5,000, 3,000, 2,000, 1,000, 750, 500, 250, 100 bp fragments, respectively. From lane 2 to 6 are five red flesh color watermelon accessions; from lane 8 to 12 are five yellowish flesh color watermelon accessions; from lane 14 to 18 are five pink flesh color watermelon accessions; from lane 20 to 24 are five white flesh color watermelon accessions.
