# Supplementary figures and images for: Fine Mapping of Lycopene Content and Flesh Color Related Gene and Development of Molecular Marker–Assisted Selection for Flesh Color in Watermelon (Citrullus lanatus)
Source: Front Plant Sci. 2019 Oct 8;10:1240. doi: 10.3389/fpls.2019.01240 (PMC6791925; doi:10.3389/fpls.2019.01240)

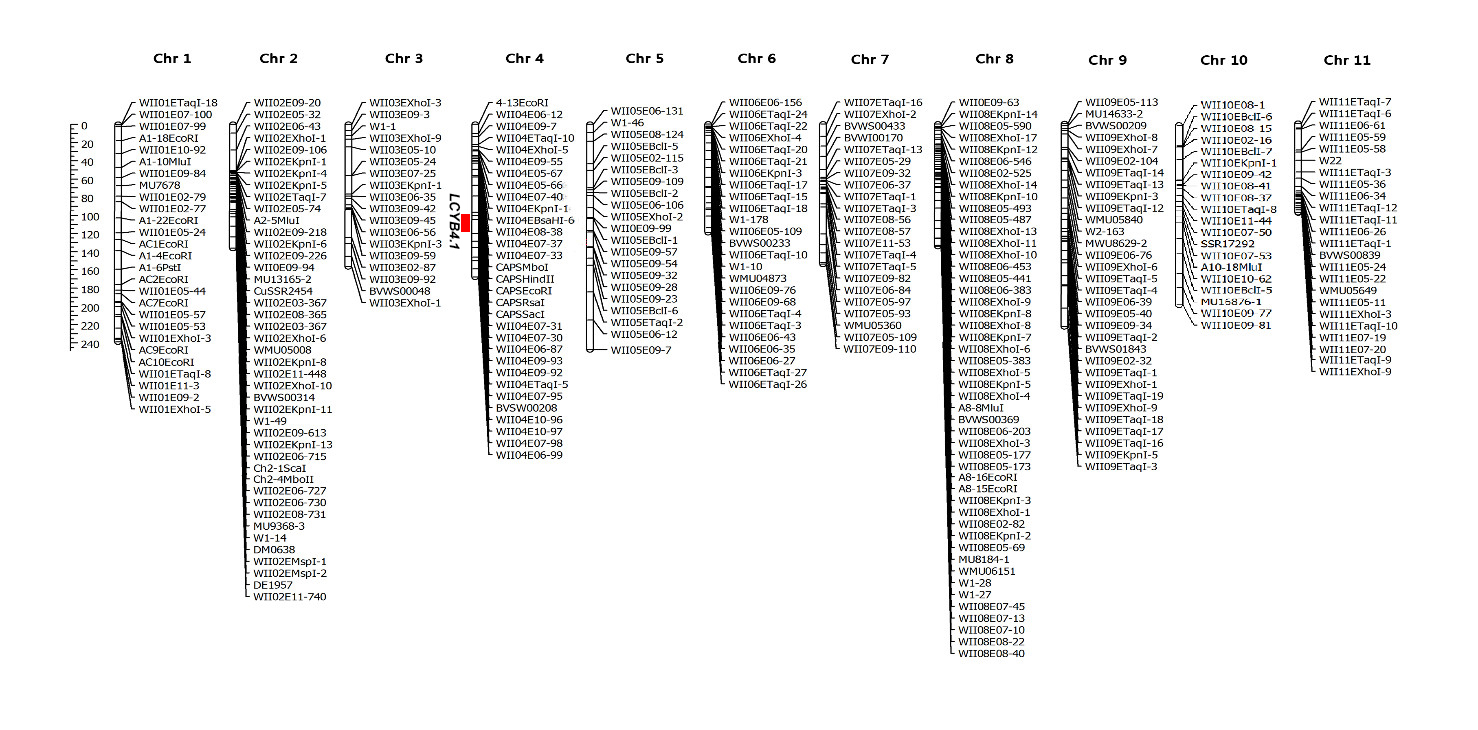

Supplement: Figure S1 — Genetic linkage map with the F2 generation (352 plants) derived from a cross between LSW-177 and COS. The shaded area on linkage group 4 indicates the location of QTLs associated with red flesh color traits (LCYB4.1). Chr: Chromosome [file Image_1.jpeg]

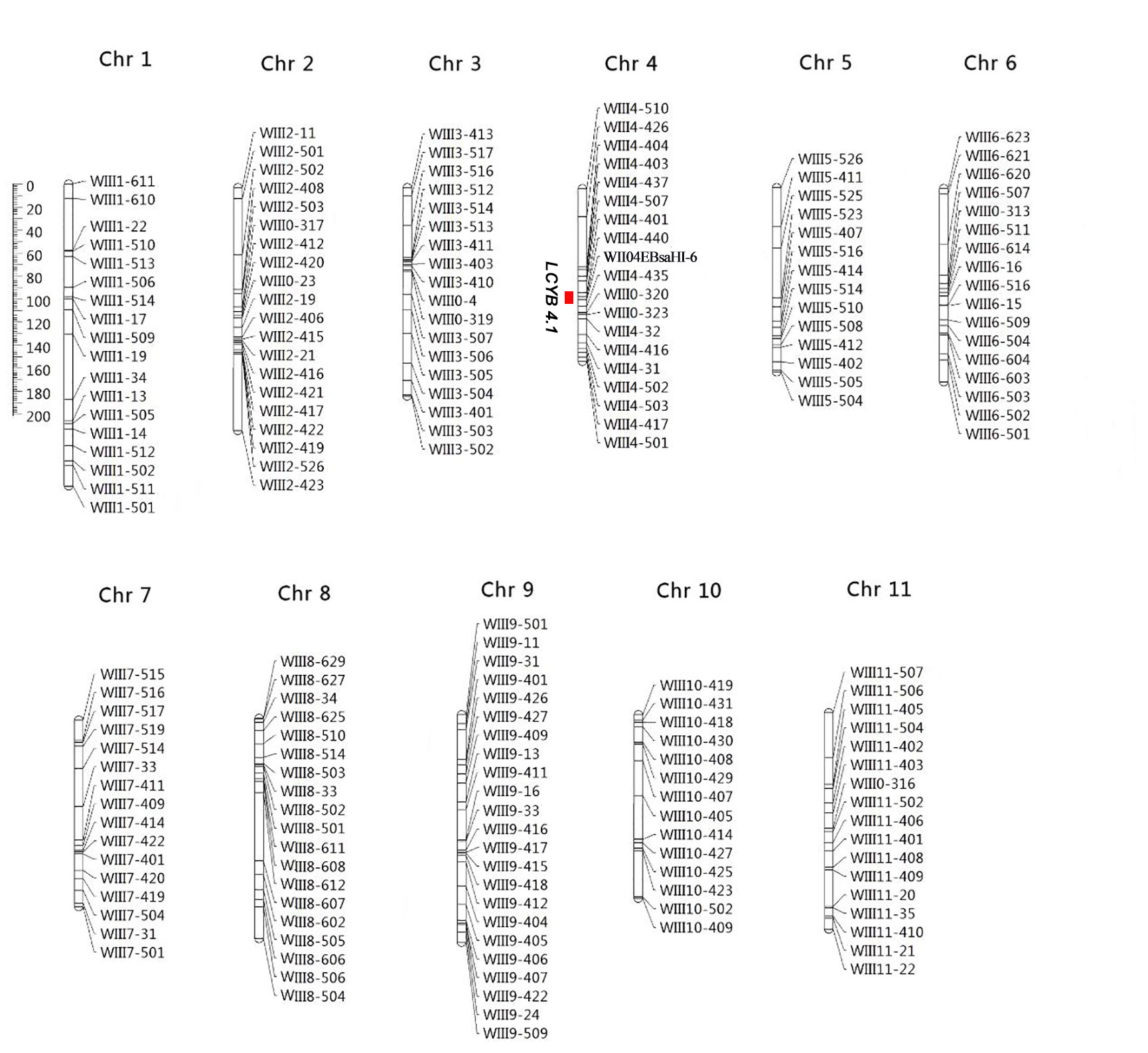

Supplement: Figure S2 — Genetic linkage map with the F2 generation (359 plants) derived from a cross between garden female and PI 186490. Chr: Chromosome The shaded area on linkage group 4 indicates the location of QTLs associated with red flesh color trait. [file Image_2.jpeg]

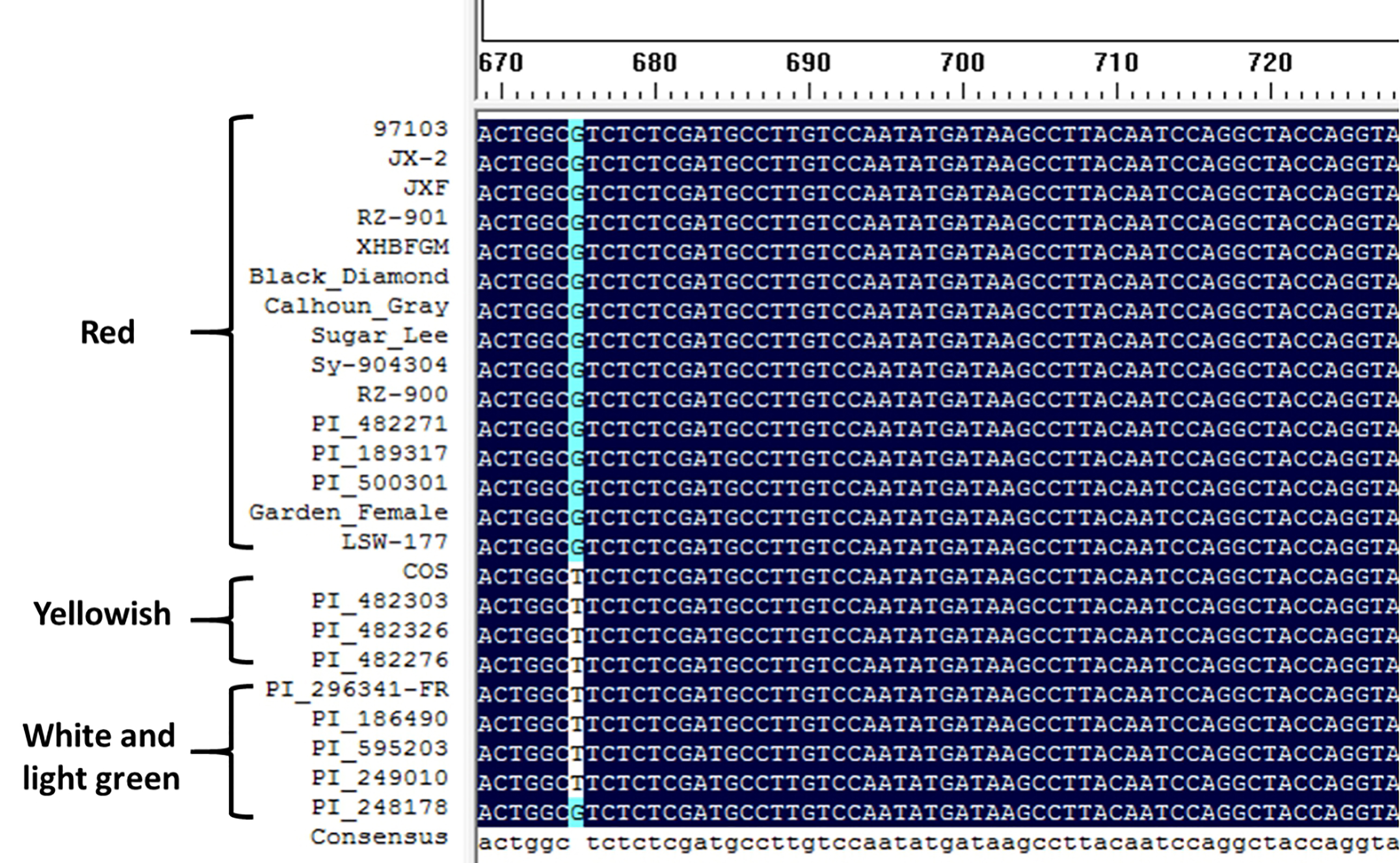

Supplement: Figure S3 — The sequence alignment of LCYB gene with 24 watermelon accessions. [file Image_3.jpeg]

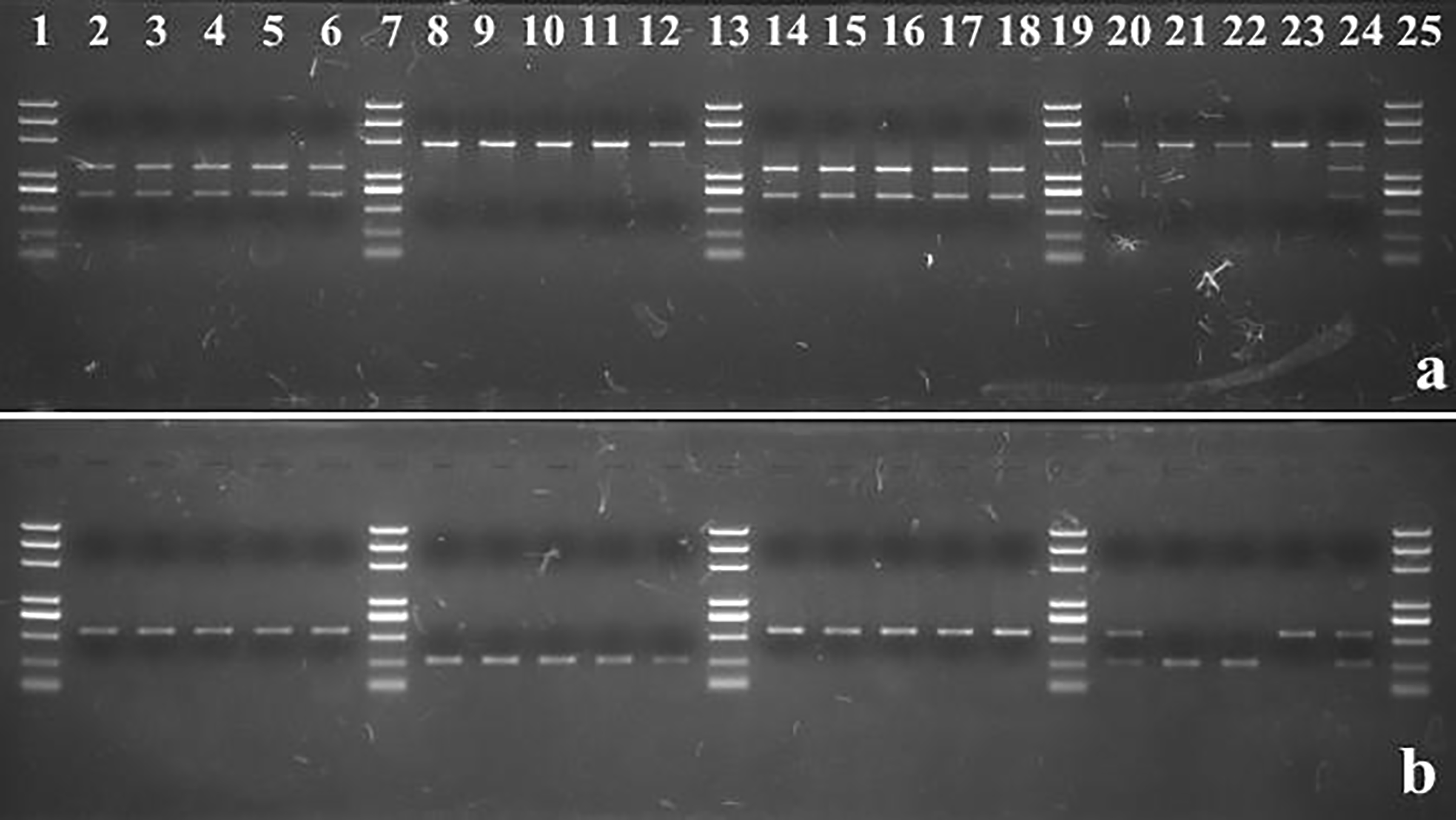

Supplement: Figure S4 — Marker analysis for red, yellowish and white flesh color watermelon accessions using CAPS marker WII04EBsaHI-6 and WII04E08-38. Supplementary Figure S4a was the genotyping results of CAPS markers WII04EBsaHI-6; figure S4b was the genotyping results of CAPS markers WII04E08-38. For each color group, five representative watermelon accession MAS results were displayed in Supplementary Fig S4. Lane 1, 7, 13, 19 and 25 is the D2000 plus DNA marker. From the top to the bottom was 5,000, 3,000, 2,000, 1,000, 750, 500, 250, 100 bp fragments, respectively. From lane 2 to 6 are five red flesh color watermelon accessions; from lane 8 to 12 are five yellowish flesh color watermelon accessions; from lane 14 to 18 are five pink flesh color watermelon accessions; from lane 20 to 24 are five white flesh color watermelon accessions. [file Image_4.jpeg]

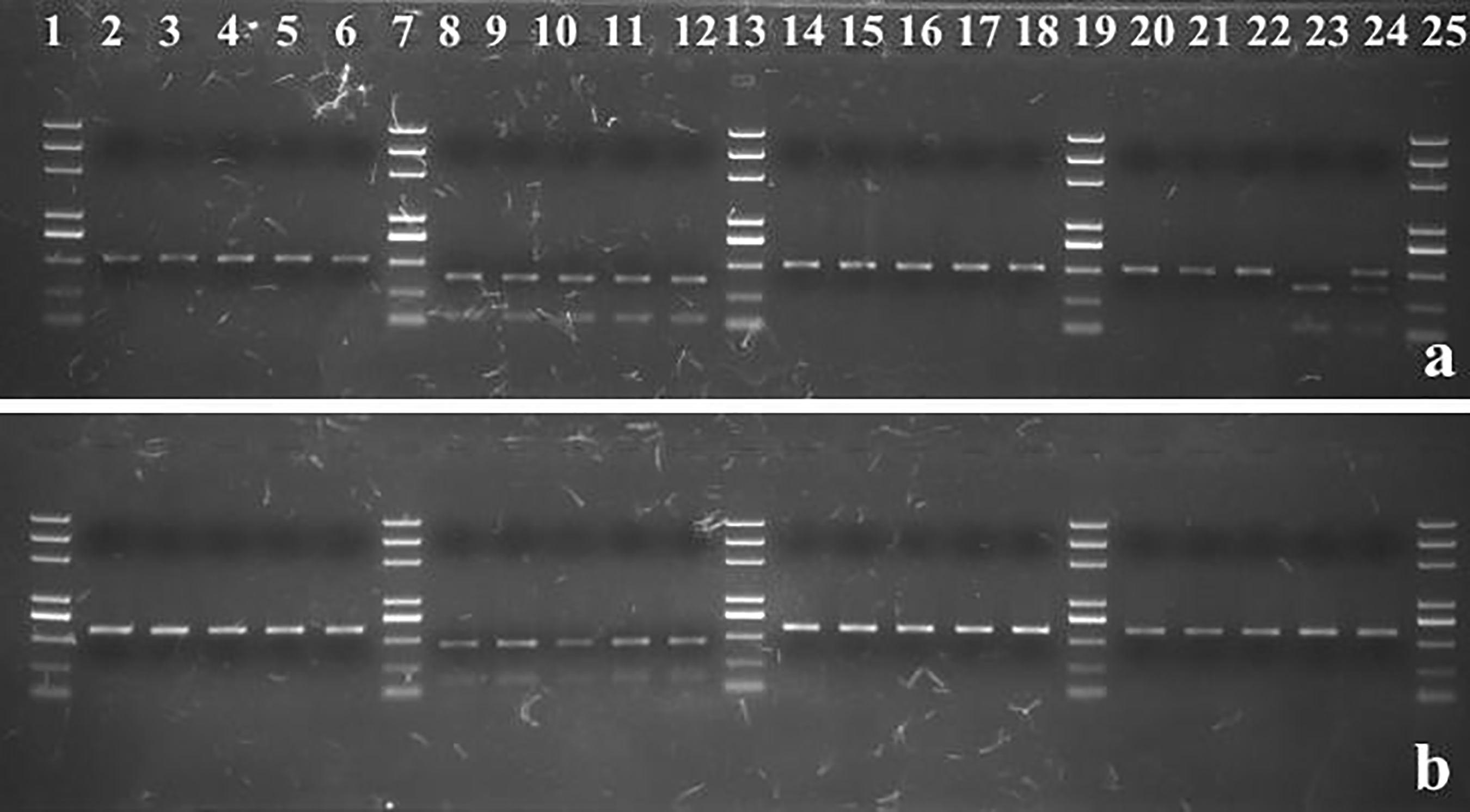

Supplement: Figure S5 — Marker analysis for red, yellowish and white flesh color watermelon accessions using CAPS marker WII04EKpnI-1 and WII04E07-40. Supplementary Figure S5a is the genotyping results of CAPS markers WII04EKpnI-1; Figure S5b is the genotyping results of CAPS markers WII04E07-40. For each color group, five representative watermelon accessions MAS results are displayed in Figure S5. Lane 1, 7, 13, 19 and 25 are the D2000 plus DNA marker. From the top to the bottom are 5,000, 3,000, 2,000, 1,000, 750, 500, 250, 100 bp fragments, respectively. From lane 2 to 6 are five red flesh color watermelon accessions; from lane 8 to 12 are five yellowish flesh color watermelon accessions; from lane 14 to 18 are five pink flesh color watermelon accessions; from lane 20 to 24 are five white flesh color watermelon accessions. [file Image_5.jpeg]
